# HMG-CoAR expression in male breast cancer: relationship with hormone receptors, Hippo transducers and survival outcomes

**DOI:** 10.1038/srep35121

**Published:** 2016-10-07

**Authors:** Anna Di Benedetto, Marcella Mottolese, Francesca Sperati, Cristiana Ercolani, Luigi Di Lauro, Laura Pizzuti, Patrizia Vici, Irene Terrenato, Abeer M. Shaaban, Sreekumar Sundara-Rajan, Matthew P. Humphries, Maddalena Barba, Valerie Speirs, Ruggero De Maria, Marcello Maugeri-Saccà

**Affiliations:** 1 Department of Pathology, “Regina Elena” National Cancer Institute, Via Elio Chianesi 53, 00144, Rome, Italy; 2 Biostatistics-Scientific Direction, “Regina Elena” National Cancer Institute, Via Elio Chianesi 53, 00144, Rome, Italy; 3 Division of Medical Oncology B, “Regina Elena” National Cancer Institute, Via Elio Chianesi 53, 00144, Rome, Italy; 4 Queen Elizabeth Hospital Birmingham and University of Birmingham, Department of Histopathology, Edgbaston, Birmingham B15 2GW, UK; 5 Leeds Institute of Cancer and Pathology, Wellcome Trust Brenner Building, University of Leeds, Leeds LS9 7TF, UK; 6 Scientific Direction, “Regina Elena” National Cancer Institute, Via Elio Chianesi 53, 00144, Rome, Italy; 7 Institute of General Pathology, Catholic University of the Sacred Heart, Largo Agostino Gemelli, 10, 00168, Rome

## Abstract

Male breast cancer (MBC) is a rare hormone-driven disease often associated with obesity. HMG-CoAR is the central enzyme of the mevalonate pathway, a molecular route deputed to produce cholesterol and steroid-based hormones. HMG-CoAR regulates the oncogenic Hippo transducers TAZ/YAP whose expression was previously associated with shorter survival in MBC. 225 MBC samples were immunostained for HMG-CoAR and 124 were considered eligible for exploring its relationship with hormone receptors (ER, PgR, AR), Hippo transducers and survival outcomes. HMG-CoAR was positively associated with the expression of hormone receptors (ER, PgR, AR) and Hippo transducers. Overall survival was longer in patients with HMG-CoAR-positive tumors compared with their negative counterparts (p = 0.031). Five- and 10-year survival outcomes were better in patients whose tumors expressed HMG-CoAR (p = 0.044 and p = 0.043). Uni- and multivariate analyses for 10-year survival suggested that HMG-CoAR expression is a protective factor (HR 0.50, 95% CI: 0.25–0.99, p = 0.048 and HR 0.53, 95% CI: 0.26–1.07, p = 0.078). Results were confirmed in a sensitivity analysis by excluding uncommon histotypes (multivariate Cox: HR 0.45, 95% CI: 0.21–0.97, p = 0.043). A positive relationship emerged between HMG-CoAR, hormone receptors and TAZ/YAP, suggesting a connection between the mevalonate pathway, the hormonal milieu and Hippo in MBC. Moreover, HMG-CoAR expression may be a favorable prognostic indicator.

Male breast cancer (MBC) is a rare disease^1^, even though its incidence has increased over the past decades^2^,^3^. Similar to other uncommon diseases, its biology is understudied despite recent efforts toward obtaining information on genomic alterations and deregulated pathways^4^,^5^,^6^,^7^,^8^,^9^,^10^. An established concept is the hormone-driven nature of the disease. First, a number of conditions that alter the hormonal milieu, such as aging, obesity, liver diseases, Klinefelter’s syndrome and testicular disorders, are linked to MBC^1^,^11^. Second, steroid receptors, such as the estrogen receptor (ER), progesterone receptor (PgR) and androgen receptor (AR), are often expressed in MBC, even more frequently than in female breast cancer (FBC)^12^,^13^. Consistently, hormone therapies are central in the medical management of MBC patients^14^,^15^,^16^.

3-Hydroxy-3-methylglutharyl-coenzyme A reductase (HMG-CoAR), the molecular target of statins, acts as the rate-limiting enzyme in the mevalonate pathway, a metabolic route that leads to the production of steroid-based hormones, cholesterol, and non-sterol isoprenoids^17^. Considering the close connection between the mevalonate pathway and hormonal stimuli, it is not surprising that HMG-CoAR expression was associated with ER positivity, anthropometric factors (e.g. obesity), increased efficacy of adjuvant tamoxifen and better survival outcomes in FBC^18^,^19^,^20^.

Nevertheless, the contribution of intratumoral HMG-CoAR to the biology of cancer is more complex, as preclinical studies uncovered a number of aberrantly regulated processes requiring the activity of the enzyme^21^. Key intermediates of the mevalonate pathway, namely farnesyl pyrophosphate (FPP) and geranylgeranyl pyrophosphate (GGPP), are central metabolites in the process of prenylation^21^. This post-translational process facilities correct membrane anchoring of a number of molecules, even including signal transduction proteins involved in oncogenic signals, such as the small GTPases Ras, Rab, Rho. Consistently with the biological importance of protein prenylation in cancer, farnesyltransferase inhibitors were developed, chiefly as Ras-targeting agents, albeit the results reported so far have been disappointing^21^.

A recently described function of HMG-CoAR refers to its control on the Hippo transducers TAZ/YAP^22^,^23^, two closely related oncoproteins acting downstream in the evolutionary conserved Hippo pathway^24^. According to this model, GGPP favors TAZ/YAP nuclear accumulation and transcription of target genes by promoting membrane localization of Rho GTPases^22^. Importantly, activation of Hippo transducers has increasingly been linked to breast cancer stem cell (CSC) function^25^,^26^,^27^,^28^, and CSC pathway analysis has been advocated as a powerful strategy for developing novel prognostic/predictive markers. Consistently, retrospective clinical studies raised the hypothesis that Hippo-related biomarkers may predict poorer outcomes in FBC^29^,^30^,^31^, and we have already provided hints that expression of TAZ/YAP and their target Connective Tissue Growth Factor (CTGF) is associated with inferior survival compared to TAZ/CTGF and YAP/CTGF negative MBC patients^32^.

Prompted by the potential “Janus-faced” role of HMG-CoAR, namely i) its connection with hormone receptors and favorable clinical outcomes observed in FBC^18^,^19^,^20^ and, on the other side, ii) the positive control that HMG-CoAR operates on a variety of oncogenic proteins, even including pathways involved in CSC fate^22^, we herein investigated HMG-CoAR expression in a large series of MBC samples. Our goals were the following: i) describing its expression pattern, ii) evaluating its association with hormone receptors (ER, PgR, AR) and Hippo transducers (TAZ/YAP plus their target CTGF), and iii) analyzing the impact of HMG-CoAR expression on survival outcomes.

## Results

### Relationship between HMG-CoAR, hormone receptors and Hippo transducers

For this retrospective study, 255 MBC samples were screened for the expression of HMG-CoAR. One hundred twenty four patients (124) were included on the basis of the eligibility criteria described in the paragraph addressing Materials and Methods. One hundred thirty one (131) patients were excluded for the following reasons: missing (N = 100) or ambiguous (N = 7) data on overall survival (OS), missing data on HMG-CoAR status (N = 17), missing data on AR status (N = 6), missing data on PgR status (N = 1).

**Figure 1:**
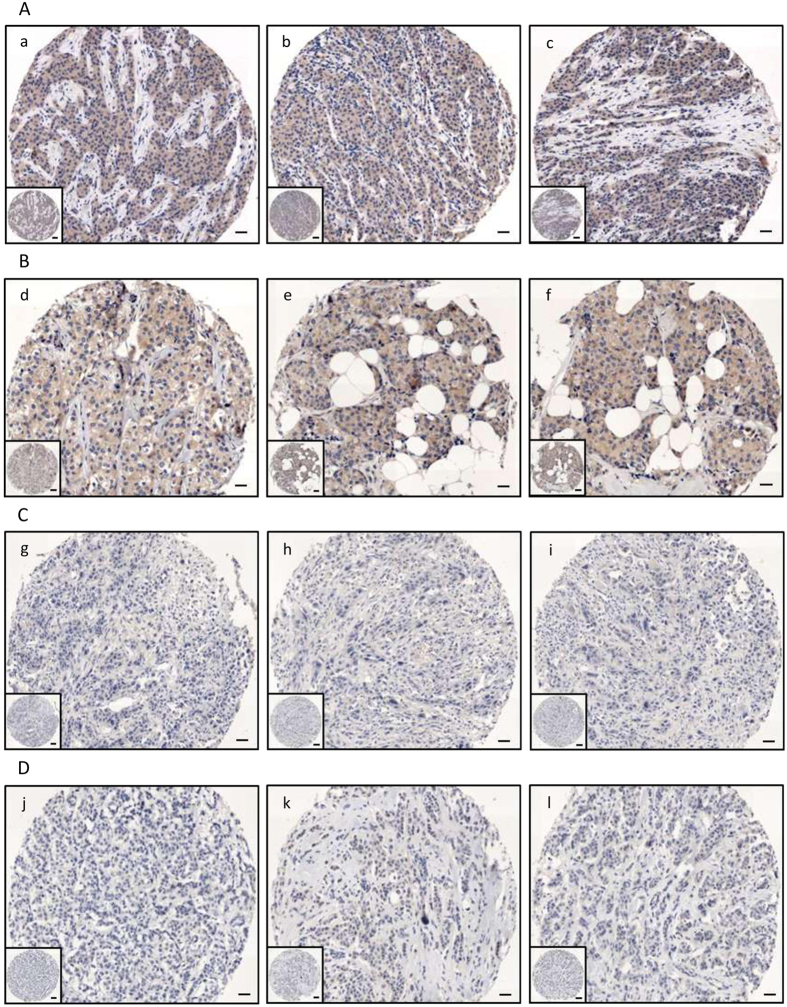
Representative examples of immunohistochemical expression of HMG-CoAR in four male breast cancer patients. Two exemplificative cases (**A,B**) of positive HMG-CoAR expression are shown, documenting homogenous cytoplasmic positivity in all the three TMA cores pertinent to each case (a–f). Two negative cases (**C,D**) are also shown (g–i; j–l). Scale bar 30 μm.

**Figure 2:**
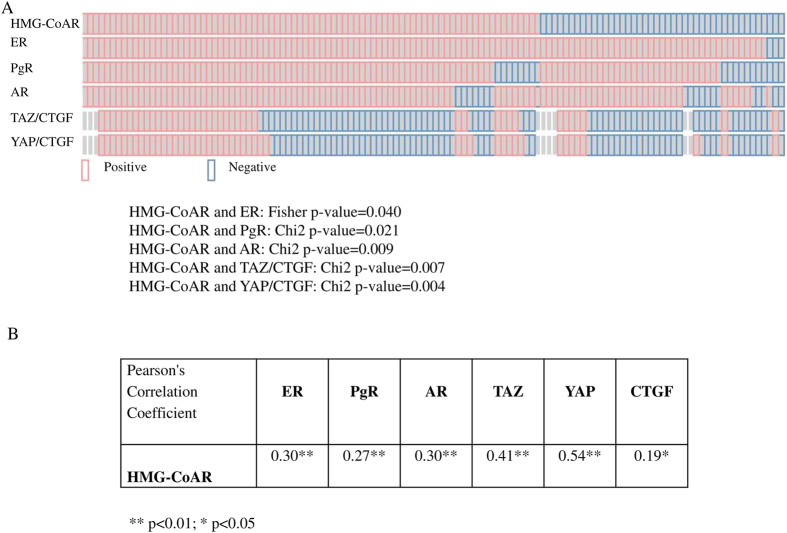
Relationship between HMG-CoAR, hormone receptors and the TAZ/CTGF and YAP/CTGF phenotypes. Associations are shown in panel (**A**) correlations in panel (**B**).

Eligible patients were diagnosed with breast cancer in the time frame between 1983 and 2009. Baseline characteristics of these patients are summarized in Table 1. Median age at diagnosis was 67.5 years. One hundred four patients (83.9%) were diagnosed with invasive ductal carcinoma (IDC) or invasive lobular carcinoma (ILC). Nodal status was described as positive in 55 patients (44.4%). 105 (84.7%) men had a ER^+^/PgR^+^ disease and Ki-67 was rated as low in 72 (58.1%) cases. When evaluated by HMG-CoAR status, 43 (34.7) patients tested negative. The formalin-fixed paraffin-embedded (FFPE) blocks of MBC patients were collected in Europe and Canada^13^. Median follow up was 183 months (95% CI 91.4–274.7). Representative immunohistochemical staining is presented in Fig. 1. When considered as a categorical variable, HMG-CoAR expression was positively associated with the expression of ER, PgR and AR (p = 0.040, p = 0.021 and p = 0.009, respectively as shown in Fig. 2, panel A). Moreover, we observed a significant positive correlation between HMG-CoAR and ER, PgR and AR (Fig. 2, panel B). Interestingly, HMG-CoAR was also positively associated with the TAZ/CTGF and YAP/CTGF phenotypes (p = 0.007 and p = 0.004, respectively; Fig. 2, panel A), and positively correlated with TAZ, YAP and CTGF (Fig. 2, panel B). Overall, our data seem to provide evidence in support of a link between the expression of HMG-CoAR and that of both hormone receptors and central actors of the Hippo signaling pathway in MBC. We did not observe any significant association between HMG-CoAR and histology, Ki-67 levels, tumor grade and nodal status (data available upon request). Similarly, the median age and range in HMG-CoAR positive and negative subjects were comparable (69 (34–84) and 66 (34–86) years, respectively).

### Prognostic Significance of HMG-CoAR expression

**Figure 3:**
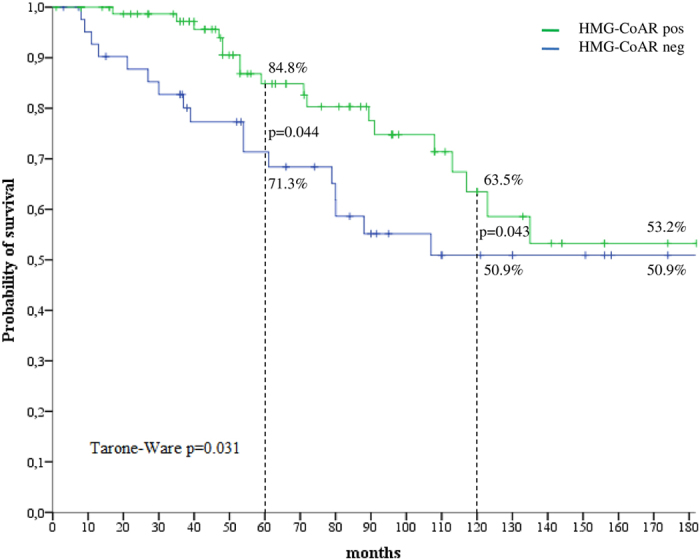
Kaplan-Meier survival curves comparing HMG-CoAR-positive and HMG-CoAR-negative cases (N = 124).

**Figure 4:**
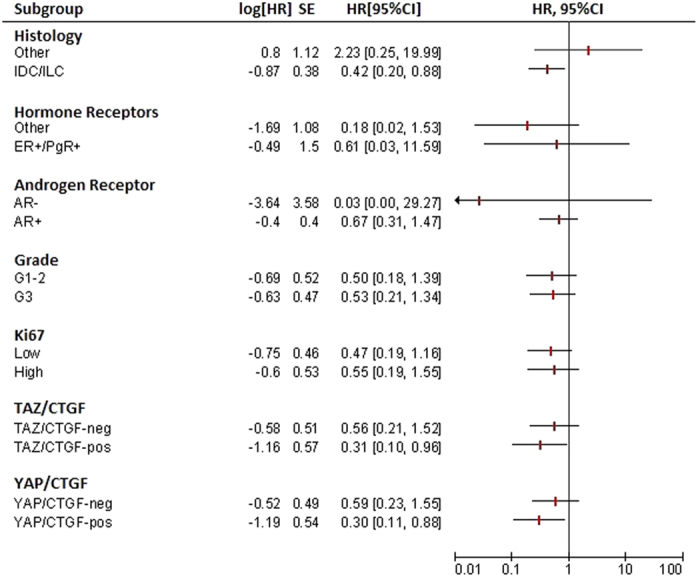
Univariate analysis and Forest plots for subgroup analysis of 10-year survival (N = 124). When considering the TAZ/CTGF and YAP/CTGF subgroups, analyses were carried out in 115 patients.

**Figure 5:**
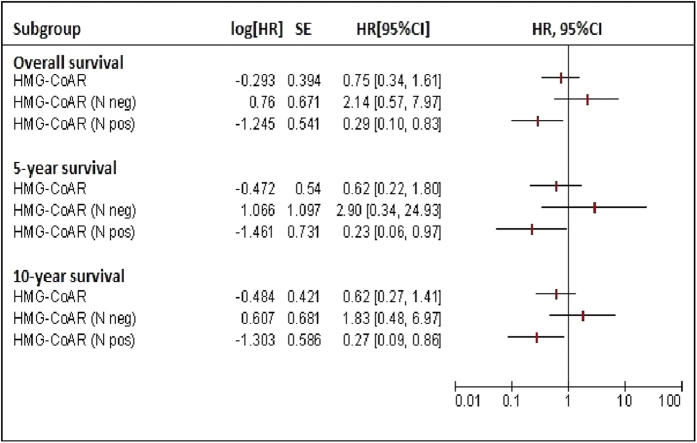
Univariate analysis and Forest plots for subgroup analysis (node-positive and node-negative) of overall, 5-, and 10-year survival (N = 91).

We next investigated the impact of HMG-CoAR on survival outcomes. OS (defined in the Materials and Methods section) was longer in patients whose tumors expressed HMG-CoAR compared with their negative counterparts (Tarone-Ware p = 0.031) (Fig. 3), as well as 5- and 10-year survival (log rank p = 0.044 and p = 0.043, respectively, Fig. 3). The univariate Cox regression analysis carried out for identifying variables impacting 10-year survival, presented in Table 2, confirmed the protective role of HMG-CoAR (HR 0.50, 95% CI: 0.25–0.99, p = 0.048), which was the only variable significantly associated with the investigated outcome. Nevertheless, we verified the role of HMG-CoAR in a multivariate model by adjusting for other plausible variables (histology, grade, hormone receptor status, and Ki-67 levels), albeit they did not test significant at the univariate assessment. As shown in Table 2, a non-significant reduction in overall mortality was observed for patients with HMG-CoAR-positive tumors comparing with those whose tumors were HMG-CoAR-negative (HR 0.53, 95% CI: 0.26–1.07, p = 0.078). In a sensitivity analysis carried out by excluding uncommon histotypes (N = 20), HMG-CoAR was fully significant in the multivariate Cox model (HR 0.45, 95% CI: 0.21–0.97, p = 0.043), as reported in Table 3. Interestingly, in subgroup analysis HMG-CoAR positivity was a protective factor in patients whose tumors harbored the TAZ/CTGF and YAP/CTGF phenotypes (HR and 95% CI were 0.31 (0.10–0.96) and 0.30 (0.11–0.88), respectively) (Fig. 4). Finally, in the subset of patients with available information on nodal involvement (N = 91), HMG-CoAR expression was associated with better survival outcomes in patients with node-positive disease, with HR and 95% CI being 0.29 (0.10–0.83), 0.23 (0.06–0.97) and 0.27 (0.09–0.86) for OS, 5- and 10-year survival, respectively (Fig. 5).

## Discussion

In the present study, we retrospectively investigated the expression of HMG-CoAR in a large cohort of MBC patients with available hormone receptor status and survival data. This cohort was previously analyzed for the expression of the Hippo transducers TAZ/YAP along with their established target CTGF, and complete data on these candidate biomarkers were available for 115 patients^32^. To our knowledge, this is the first report addressing the mevalonate pathway and its connection with established and emerging oncogenic signals in MBC. Overall, our data suggest that, on the one hand, HMG-CoAR acts in different oncogenic networks and, on the other hand, its expression might represent a favorable prognostic indicator in MBC, similar to earlier reports in FBC^18^,^19^,^20^.

This study has some limitations. In interpreting our results, it is worth mentioning that we tested the impact of HMG-CoAR on survival outcomes that also included cancer-unrelated events. This is due to lack of complete information pertinent to the cause of death (deaths: 38; MBC-related deaths: 13 (34.2%); deaths from unknown cause: 25 (65.8%), as well as detailed information on adjuvant therapy. In a greater detail, 8 MBC-related deaths were recorded in the HMG-CoAR-positive group and 5 in the HMG-CoAR-negative group (Chi2 p = 0.762). When considering deaths from any cause, 20 events were recorded in the HMG-CoAR-positive group and 18 in the HMG-CoAR-negative group (Chi2 p = 0.048). Nevertheless, the use of intermediate time-points (e.g. 5- and 10-year survival) in a disease characterized by a long natural history, comparable to that of luminal-type FBC^14^, may mitigate the partial unavailability of cancer-specific events. Second, nodal involvement was not considered in the uni- and multivariate models given that, despite our best efforts, this information was retrieved only for 91 patients. In this subset, HMG-CoAR expression was associated with better survival outcomes in the node-positive disease. However, given the limited number of patients included in subgroup analyses, presented in Figs 4 and 5, these results require further validation in adequately powered studies. It is worth mentioning the limited availability of data on adjuvant treatments, which were only available for a restricted number of patients. This is extremely common when relying on clinical series, particularly when data and biological samples are provided by several participating centers. Nevertheless, it is worth considering that a retrospective study shown no benefit from treatment with aromatase inhibitors compared with tamoxifen, which remains the treatment of choice in the adjuvant setting for hormone receptor-positive MBC^33^. Thus, adjuvant therapy for MBC patients, which mostly consists in hormone therapies in consideration of the frequent expression of hormone receptors, has remained substantially unvaried over the past decades^14^.

In our opinion, our study provides some interesting insights beyond the suggestion of a prognostic significance of HMG-CoAR expression. First, our results place HMG-CoAR in the hormonal network that fuels the genesis of MBC, as documented by the significant positive relationship between HMG-CoAR and hormone receptor status (ER, PgR, AR). These data open up to a scenario where systemic and local (intratumoral) endocrine and metabolic factors participate in recreating a “hormone-rich” background. Three observations explain this: i) in FBC, HMG-CoAR expression was positively associated with overweight^18^, ii) in turn, obesity was designed as a risk factor for MBC as a consequence of the age-related increase in both aromatase activity and fat mass, which lead to an excess of estrogens^11^,^34^,^35^,^36^, and iii) molecular studies documented intratumoral aromatase expression and elevated levels of 17β-estradiol in MBC tissues^37^,^38^, and both these observations may be connected with intratumoral activation of the mevalonate pathway.

In recollecting this evidence, it is plausible that intratumoral HMG-CoAR expression represents an important source of hormonal stimuli that drive MBC carcinogenesis and whose activity, both in cancer cells and in other tissues^39^, is enhanced by the underlying metabolic status of these patients (e.g. obesity and related metabolic disorders). On this basis, we suggest to carry out extensive metabolic characterization of MBC patients in future research that, beyond body mass index, should envision additional tests such as dual-energy X-ray absorptiometry (DEXA) and lipidic profile with 27-hydroxycholesterol (27HC) assessment, as 27HC acts as a selective ER modulator inducing proliferation of ER-expressing breast cancer cells^40^,^41^,^42^. Moreover, considering the connection between obesity and MBC, our data support the hypothesis that statins may have anticancer properties, especially in HMG-CoAR-expressing MBC patients. To this end, statins were already found to produce anti-proliferative effects (Ki-67 reduction) in a pre-surgical, window-of-opportunity FBC trial, further corroborating the involvement of the mevalonate pathway in hormone-driven breast cancer^43^.

Next, the positive relationship observed between HMG-CoAR and Hippo transducers provides further ground to the preclinically described control on TAZ/YAP operated by the mevalonate pathway. Interestingly, while the co-expression of TAZ/YAP and CTGF was previously associated with unfavorable survival outcomes in MBC^32^, HMG-CoAR appeared to be a protective determinant in the TAZ/CTGF- and YAP/CTGF-positive backgrounds, as revealed by subgroup analysis. The following, potentially connected, explanations may justify these observations. First, beyond metabolic cues, further Hippo regulatory branches supposedly operate in MBC. This is not surprising when considering the complexity of Hippo biology and the number of molecular inputs that modulate TAZ/YAP activity, such as mechanotransduction, cell density, cell-cell adhesion mechanisms, apical-basal polarity factors, hypoxia and the Wnt/β-catenin pathway^44^. Second, in the subset of MBC with active TAZ/YAP-driven transcription, HMG-CoAR expression may delineate a further fraction of tumors at a prevalent endocrine-metabolic asset, and be possibly endowed with less aggressive molecular traits.

Our results suggest that HMG-CoAR expression in MBC patients is a potential prognostic marker that deserves further attention. Moreover, our findings add a further piece to the biology of MBC, overall linking metabolic cues, endocrine stimuli and stem cell pathways. Finally, we would like to encourage investigators engaged in MBC research to reconsider survival outcomes in light of history of statin use and HMG-CoAR expression, in order to collect preliminary evidence on the therapeutic potential of cholesterol-lowering agents for MBC patients.

## Material and Methods

### Study Participants and procedures

Biological samples from 255 histologically confirmed MBC patients, represented on tissue microarrays (TMAs), were immunohistochemically evaluated for the expression of HMG-CoAR. Eligibility criteria for inclusion were the following: i) complete data on HMG-CoAR, ii) complete data on hormone receptors (ER, PgR, AR), and iii) availability of survival data. On this basis, 124 patients were considered eligible. The information related to the expression of TAZ, YAP and CTGF was available for 115 patients. The biological samples related to patients fulfilling the inclusion criteria were immunostained in 2016. Complete data pertinent to post-surgical therapy were not extensively available; in this series adjuvant therapy mostly consisted of tamoxifen. Information related to adjuvant therapy was available only for 25 patients, of whom 19 received tamoxifen, 5 anastrozole and 1 did not receive any adjuvant treatment. Nodal status was available for 91 patients, and this subset was independently analyzed.

TMAs were built from FFPE material, as already detailed^13^. The immunohistochemical assessment of HMG-CoAR was performed in FFPE tissues using the polyclonal antibody anti-HMG-CoAR (HPA008338, Sigma-Aldrich, St. Louis, USA) at the dilution of 1:150, as reported in a clinical trial investigating high-dose atorvastatin in FBC^43^.

HMG-CoAR expression was assessed both in terms of staining intensity (0: negative, 1+: weak, 2+: moderate, 3+: strong) and percentage of cytoplasmic-expressing cells (0–100%). Samples were considered positive if ≥30% of neoplastic cells exhibited a distinct cytoplasmic immunoreactivity of any intensity. The logic behind the use of this cutoff is detailed in the “statistical analysis” section. Immunoreactivity was evaluated independently by two investigators (ADB and MM) blinded to treatment outcome. Hormone receptor immunoreactivity was scored using the Allred system, and considered positive when >2. The Allred score (ranging from 0–8) was obtained by summing the score assigned to the percentage of tumor-expressing cells (0: No cells positive; 1: ≤1% of positive cells; 2: 1 to 10% of positive cells; 3: 11 to 33% of positive cells; 4: 34 to 66% of positive cells; 5: 67 to 100% of positive cells)^13^,^45^ and the score assigned to staining intensity (0: negative; 1: weak; 2: intermediate; 3: strong). Regarding TAZ, YAP and CTGF, samples were considered to harbor active TAZ/YAP-driven transcription when they co-expressed TAZ and CTGF (TAZ/CTGF) or YAP and CTGF (YAP/CTGF)^32^.

This retrospective study was conducted in accordance with the Declaration of Helsinki and approved by the Institutional Review Board of the “Regina Elena” National Cancer Institute of Rome and by the Leeds (East) Research Ethics Committee (06/Q1205/156). As already specified, informed consent was not required as samples were anonymized to the research team^13^.

### Statistical analysis

The Pearson’s Chi-squared test of independence (2-tailed) or the Fisher Exact test were used to assess the relationship between categorical variables. The Pearson’s correlation coefficient was used to investigate the correlation between HMG-CoAR and the following biomarkers: ER, PgR, AR, TAZ, YAP, and CTGF.

Receiver operative characteristics (ROC) curves were used to establish the optimal cut-off values for HMG-CoAR in reference to survival outcomes with the highest sensitivity and specificity. The Youden’s index was then computed to identify the cut-off values that maximized the difference in sensitivity and specificity and detected true-positive and false-positive subjects across the different cut-off points. With this approach, HMG-CoAR was considered positive when expressed in ≥30% of tumor cells.

OS was calculated as the time from diagnosis to death due to any cause. Five- and 10-year survival were calculated as the time from diagnosis to death due to any cause within a 5- and 10-year timeframe, respectively. Survival curves were estimated with the Kaplan-Meier method and compared by log-rank test or Tarone-Ware test. Uni- and multivariate Cox proportional hazard models were built to identify variables impacting 10-year survival, and reported as Hazard Ratios (HR) and 95% Confident Interval (CI). Multivariate models included histology, grade, hormone receptor status (ER/PgR), Ki-67 levels and HMG-CoAR expression.

Random hot deck (RHD) imputation was used for the treatment of missing values (histology: 1/124, tumor grade: 2/124, Ki-67 levels: 4/124)^46^. By matching for auxiliary variables (histology, tumor grade, ER, PgR and Ki-67, depending upon the nature of the missing value) pools of donors were identified and a donor was randomly extracted. Then, the value of interest was assigned to the recipient. Statistical tests were two-sided, and p values below.05 were considered statistically significant. Statistical analyses were carried out using SPSS software (SPSS version 21, SPSS Inc., Chicago, IL, USA).

## Additional Information

**How to cite this article**: Di Benedetto, A. *et al*. HMG-CoAR expression in male breast cancer: relationship with hormone receptors, Hippo transducers and survival outcomes. *Sci. Rep*. **6**, 35121; doi: 10.1038/srep35121 (2016).

## Figures and Tables

**Table 1:** Baseline characteristics of MBC patients included in this study (N = 124).

Characteristics		N (%)
**Age at diagnosis**α	Median (min-max) [IQ range]	67.5 (34–86) [59–75]
*Histology	IDC/ILC	104 (83.9)
	Other**	20 (16.1)
*Grade	G1-2	65 (52.4)
	G3	59 (47.6)
**Nodal status**	Negative	36 (29.0)
	Positive	55 (44.4)
	Unknown	33 (26.6)
**Hormone Receptors**	ER^+^/PgR^+^	105 (84.7)
	Other•	19 (15.3)
*Ki-67	Low	72 (58.1)
	High	52 (41.9)
**HMG-CoAR**	Neg	43 (34.7)
	Pos	81 (65.3)

^α^Computed in 106 patients.

^*^Variables with missing values. The number of cases with missing values by variable of interest was as follows: histology: 1/124, tumor grade: 2/124, and Ki-67 levels: 4/124. The missing values were replaced using the random hot deck method (Materials and Methods).

^**^Other: Adenocarcinoma (N = 3), Intraductal Papillary Carcinoma (N = 2), Papillary Carcinoma (N = 6), Mucinous carcinoma (N = 2), Mixed (N = 4), Medullary (N = 1), Micropapillary (N = 1), Tubular (N = 1).

^•^Other: ER^+^/PgR^−^ (N = 16), ER^−^/PgR^−^ (N = 3).

Abbreviations: IDC: Invasive Ductal Carcinoma, ILC: Invasive Lobular Carcinoma.

**Table 2:** Univariate and multivariate Cox regression analysis for 10-year survival (N = 124).

		Univariate Cox		Multivariate Cox§	
		HR (95% CI)	p-value	*HR (95% CI)	p-value
**Histology**	**IDC/ILC vs. other**	1.03 (0.40–2.66)	0.955	0.92 (0.35–2.42)	0.871
**Grade**	**G3 vs. G1-2**	1.53 (0.77–3.03)	0.225	1.41 (0.68–2.94)	0.356
**Hormone Receptors**	**ER**^**+**^**/PgR**^**+**^ **vs. other**	0.70 (0.30–1.62)	0.406	0.87 (0.37–2.08)	0.758
**Ki-67**	**High vs. Low**	1.10 (0.55–2.21)	0.777	1.01 (0.49–2.08)	0.985
**HMG-CoAR**	**Pos vs. Neg**	0.50 (0.25–0.99)	0.048	0.53 (0.26–1.07)	0.078

^*^HR and 95% CI were calculated for each variable, mutually adjusted for all other covariates.

^§^Adjusted for: Histology, Grade, Hormone Receptors, and Ki-67.

**Table 3:** Univariate and multivariate Cox regression models for 10-year survival in IDC/ILC patients (N = 104).

		Univariate Cox regression model		Multivariate Cox regression model§	
		HR (95% CI)	p-value	*HR (95% CI)	p-value
**Grade**	**G3 vs. G1-2**	1.44 (0.68–3.05)	0.336	1.33 (0.60–2.96)	0.487
**Hormone Receptors**	**ER**^**+**^**/PgR**^**+**^ **vs. other**	0.56 (0.24–1.32)	0.184	0.72 (0.29–1.78)	0.474
**Ki-67**	**High vs. Low**	1.08 (0.51–2.28)	0.843	1.03 (0.46–2.30)	0.949
**HMG-CoAR**	**Pos vs. Neg**	0.42 (0.20–0.89)	0.023	0.45 (0.21–0.97)	0.043

^*^HR and 95% CI were calculated for each variable, mutually adjusted for all other covariates.

^§^Adjusted for: Grade, Hormone Receptors and Ki-67.
